# Cerebellar Mutism Syndrome in a Patient With Hypertensive Urgency and Ischemia: A Case Report

**DOI:** 10.7759/cureus.75368

**Published:** 2024-12-09

**Authors:** Shounak Ghosh, Bertrand Liang

**Affiliations:** 1 Department of Neurology, St. Joseph Medical Center, Stockton, USA; 2 Department of Neurology, University of Colorado, Colorado Springs, USA

**Keywords:** cerebellar mutism, neurology and critical care, outcomes of hypertensive emergency, posterior fossa, stroke

## Abstract

Cerebellar mutism syndrome (also known as posterior fossa syndrome) has been mostly seen in pediatric patients after surgery for neoplastic disease and is characterized by mutism, with variable symptoms such as emotional lability, ataxia, apraxia, and hypotonia. While the mechanism is not precisely defined, it is thought to result from disconnections between the cortical and cerebellar brain networks. Presentation in adult patients is rare, with various etiologies including posterior fossa ischemia, hemorrhage, and tumors being most reported. We report a case of adult-onset posterior fossa syndrome in a woman with a previous right hemisphere cerebellar stroke, in the context of contralateral cerebellar involvement and hypertensive urgency. The etiology, clinical presentation and course, and management of posterior fossa syndrome are discussed.

## Introduction

Cerebellar mutism syndrome (aka posterior fossa syndrome) is a rare condition associated with posterior fossa surgery in children, often with the diagnosis of cancer (usually medulloblastoma)[1]. The syndrome is manifest by mutism of speech subsequent to cerebellar involvement and can be associated with emotional lability, ataxia, apraxia, and hypotonia [2]. It has been hypothesized that midline involvement of the cerebellum may be a potential risk factor for the development of the syndrome [3]. Usually, the symptoms, particularly the mutism, resolve over days to months with an associated improvement of other cerebellar signs [4].

The syndrome has been reported in adults as well, but far less frequently. Zedde et al. [5] reported a case of cerebellar hemorrhage associated with cerebellar mutism syndrome and reviewed the literature to describe adult cases. In their seminal review, etiologies included basilar artery occlusion, primary tumors of the posterior fossa, metastatic lesions within the posterior fossa, and ischemic and hemorrhagic disease of the cerebellum [5]. We report a novel case of cerebellar mutism syndrome in an adult with a prior history of cerebellar stroke and presumed contralateral cerebellar hemisphere ischemic involvement in the setting of hypertensive urgency, which resolved over approximately a day with active therapy for hypertension and vascular disease.

## Case presentation

A 56-year-old right-handed woman, with a history of a right cerebellar stroke three years prior to admission, as well as hypertension, type 2 diabetes, hyperlipidemia, obstructive sleep apnea, and obesity, presented with dizziness and left arm “weakness.” The patient's spouse noted that he was called by the patient in the afternoon due to her feeling poorly, manifesting as an episode of double vision and dizziness which was associated with vomiting. Instead of bringing the patient back home, he brought the patient to the emergency department for further evaluation. 

The patient was noted to not have any recent illnesses but did have some issues of recent low back strain from heavy lifting. The patient denied shortness of breath, chest pain, abdominal pain, loss of vision, or loss of consciousness. She did complain of fatigue. Medications included apixaban, fluticasone propionate, insulin, hydralazine, losartan, metformin, metoprolol tartrate, rosuvastatin, and ondansetron.

The patient was evaluated by the stroke team. The National Institutes of Health (NIH) Stroke score upon initial evaluation was 0. Cardiac, pulmonary, and abdominal exams were noncontributory. During the evaluation, the patient’s initial blood pressure was 182/86. However, in the ensuing 30 minutes, the patient became more confused, and initially began only to be able to speak in one or two-word sentences, and then became mute with no spoken speech to questions. The patient was emergently re-evaluated and found to be grossly ataxic, unable to stand, with left-sided ataxia in the upper extremity. Her blood pressure at the time was 206/126. Other vital signs at this time included a temperature of 37.3 degrees Celsius, pulse of 118, SpO2 96%, and respiratory rate of 16.

A mental status examination showed the patient to be alert, but mute; she would follow commands slowly, but accurately. Initially, she would be docile, but became angry during the examination without provocation, then docile once again. This occurred several times during the examination. Cranial nerve evaluation revealed normal pupillary response to light and accommodation, full yet saccadic extra-ocular movements, with bilateral end gaze nystagmus, intact facial sensation to light touch, no facial droop with normal eye closing, and intact neck flexion, with midline tongue protrusion and uvula elevation with symmetric shoulder shrug. Motor examination revealed no focal weakness, with normal bulk and decreased resistance to passive manipulation in the right upper extremity and bilateral lower extremities; sensory examination was intact to light touch. Reflexes were 1+ and symmetric with flexor plantar responses. Coordination examination showed ataxia with dysmetria during finger-finger-nose testing and fast finger movements in the left upper extremity; the patient required a two-person assist to stand and revealed an ataxic gait.

Laboratory studies demonstrated hypokalemia and elevated serum glucose; complete blood count revealed an elevated WBC count. Table 1 shows the complete laboratory values for the patient.

**Table 1:** Laboratory values on admission

Parameters	Reference values	Results
Sodium	136-145 mmol/L	135
Potassium	3.5-5.1 mmol/L	2.9
Chloride	96-116 mmol/L	103
Co2	20-30 mmol/L	25
Blood urea nitrogen	6-24 mg/dL	17
Creatinine	0.65-1.36 mg/dL	1.05
Glucose	70-99 mg/dL	234
Calcium	8.3-10.1 mg/dL	9.7
White blood cell count	3.7-11.8 10*3/µL	14.3
Hemoglobin	13.4-18.0 g/dL	16.6
Hematocrit	41.4-53.0 %	47.8
Platelets	150-400 10*3/µL	229
Neutrophils percent	51-64 %	87
Monocytes percent	0-8%	7
Eosinophils percent	0-4%	0
Lymphocytes	21-61%	6

**Figure 1:**
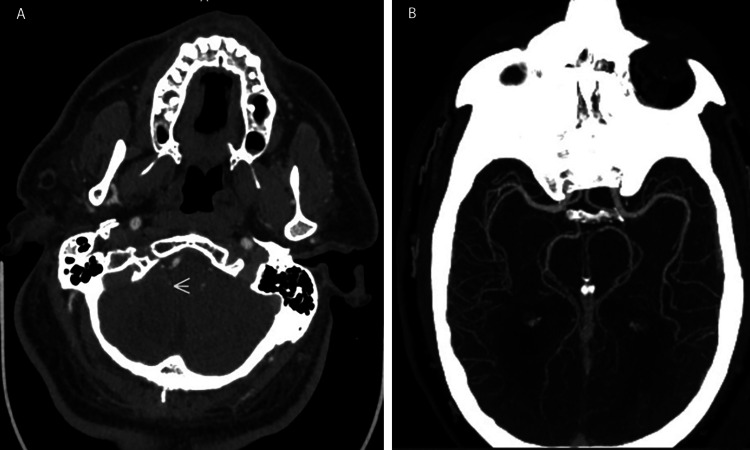
CT scans. A. CTA shows the area affected by the right proximal posterior inferior cerebellar artery occlusion (white arrows). B. AI algorithm (Viz.ai., San Francisco, CA) CTA demonstrating no large vessel occlusion.

CT scan revealed no acute abnormality. CTA showed a proximal posterior inferior cerebellar artery (PICA) occlusion (Figure 1), similar to that noted three years previously. 

Given the acute change in symptoms, the patient was treated with tenecteplase (TNK) after blood pressure was reduced with labetalol. Subsequently, the patient’s blood pressure became significantly labile, requiring both fluid boluses alternating with suppressors for control. Over the ensuing 12 hours, the patient’s blood pressure stabilized at <140/88. During this time, the patient was initially mute, but gradually began to speak with one and two-word sentences; after approximately eight hours, the patient began speaking in normal although hesitant speech, associated with decreased ataxia in the left upper extremity. Subsequently, about four hours later, the patient was evaluated by the physical therapy department, who noted the patient with normal speech, and no longer being ataxic either in the left upper extremity, or upon standing.

**Figure 2:**
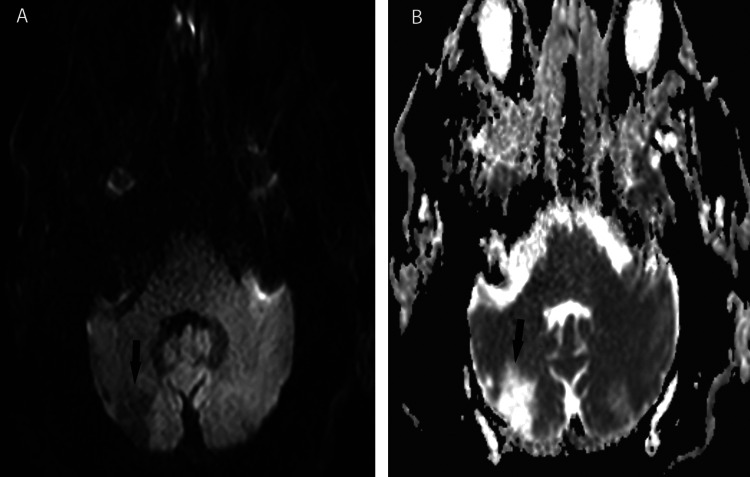
Follow up MRI A. Trace image. B. ADC image. Arrows show chronic right cerebellar hemisphere stroke, with no evidence of acute ischemia.  Hyperdense areas on the ADC image in the left cerebellar hemisphere were considered artifact as they did not appear on other MRI sequences.

A follow-up MRI scan (Figure 2) revealed the chronic right cerebellar infarct, but no evidence of acute ischemia, with mild to moderate cerebral white matter disease consistent with chronic small vessel ischemia. An area of hyperintensity on ADC images in the left cerebellar hemisphere was thought to be an artifact by radiology as it was not present on other sequences. The patient herself noted she had been “really confused” during the previous 24-36 hours, with being “uncoordinated” on the left side with problems walking, and a “strange” inability to speak. The patient was subsequently discharged two days later, asymptomatic, with the diagnosis of transient ischemic attack/aborted stroke within the posterior fossa with hypertensive urgency.

## Discussion

Cerebellar mutism syndrome is one typically associated with children who have undergone surgery for posterior fossa tumors [2]. It is much less common in adults, with several patients reported with cerebellar stroke [5-7]. In addition, in the small number of cases of adults reported, cerebellar hemorrhage affecting bilateral cerebellar hemispheres has been noted, as well as other myriad etiologies including neoplastic lesions [5,6]. The localization has been investigated with sophisticated imaging such as TRACULA (TRActs Constrained by UnderLying Anatomy), with the hypothesis of cerebello-frontal disconnection and/or damage of the brain network between the occipital lobe, cingulate gyrus, and cerebellum being involved [8]. Others have suggested the dentate-thalamo-cortical pathway plays a role in the development of the syndrome [9]. Of note, many of the cases suggest bilateral involvement of the cerebellum and/or vermis in those patients developing cerebellar mutism syndrome [10]. 

In our case, there are several unique aspects. The patient has already had an ischemic lesion in the right cerebellar hemisphere, and the acute changes in behavior, from conversant and dizziness to mutism and ataxia, prompted an acute re-evaluation of the patient, with the administration of TNK to abort a potential for acute stroke. The patient’s ataxia was localized more to the left cerebellar hemisphere, given the left upper extremity findings, with the ataxic gait suggestive of bilateral cerebellar and/or vestibulocerebellar involvement. The concern at the time was the patient was having new ischemia in the posterior fossa. Moreover, the increase in blood pressure was considered to be an exacerbating factor, with the potential for hypertensive urgency either affecting or aggravating the ischemia and/or an anamnestic syndrome of the right cerebellar stroke. The presumed bilaterality of the cerebellar involvement (the right from a previous stroke, the left and potentially vestibulocerebellar noted on clinical examination) is consistent with the hypothesis of cerebellar mutism syndrome requiring both cerebellar hemispheres [11]. 

Interestingly, our patient had high blood pressure readings associated with the clinical findings noted. With the stabilization of her blood pressure below 140/88 for several hours, despite allowance for permissive readings to a systolic of 180 as per protocol of patients receiving TNK, her clinical syndrome gradually resolved, with the return of spontaneous speech, as well as resolution of the left upper extremity ataxia and gait issues. We hypothesize the patient may have been having a transient ischemic attack involving the posterior fossa, particularly the left cerebellum, which was potentially worsened by acute changes in blood pressure. Moreover, the patient may have also been having a recrudescence of her previous stroke, in combination with contralateral ischemia. While it is unclear whether the administration of TNK aborted an ischemic stroke, the absence of any ischemic damage on MRI suggested a more transient episode in any event, without permanent sequelae, consistent with the clinical findings. Others have found transient mutism in a patient with vasospasm, as well [3].

The duration of the patient’s symptoms overall was about 24-28 hours, during which active management of the patient for ischemic disease and blood pressure control took place. In children, the cerebellar mutism syndrome duration has been found to be diverse, from one day to four months [12]. There is very limited data in adults on the duration of the syndrome, reflecting the rarity of the disorder in this age group as well as the different etiologies associated [5]. In our case, the duration of the symptoms may have reflected the active management of the clinical scenario, associated with both the mechanism of the syndrome (e.g. ischemia and recrudescence) and the correction thereof allowing for relatively prompt resolution of her mutism with ataxia. Further localization studies with higher resolution imaging (e.g. 7.0 Tesla MRI) would be interesting in further defining disconnections in such patients.

The diagnosis of cerebellar mutism syndrome has been variable since its original description (see Stoodley and Schmahmann [13] for a comprehensive review). More recently, an expert survey was performed to define diagnostic criteria in order to homogenize diagnosis as well as develop better research and treatment options [14]. The proposed diagnostic criteria include four criteria in order to effect the diagnosis of cerebellar mutism syndrome (see Table 2). Notably, an acquired cerebellar injury, combined with mutism, is alone considered sufficient for a diagnosis of the disorder. Clinically, our patient fulfilled these criteria on the basis of the left upper extremity and gait ataxia suggesting ischemic involvement of the left cerebellum, and the previous right cerebellar hemisphere stroke, with the development of mutism. Interestingly, our patient also fulfilled the criterion for irritability (the bursts of anger alternating with docility) and, as noted, ataxia. Hence, our case with respect to the expert survey with diagnostic criteria, fulfilled all criteria for the diagnosis of cerebellar mutism syndrome.

**Table 2:** Proposed diagnostic criteria for posterior fossa syndrome The proposed diagnostic criteria have been adapted from Wickenhauser et al. [14].

Criterion	Properties
Criterion A	Acquired cerebellar injury (e.g. post-surgical or stroke-related), with symptoms in Criteria B, and C or D, emerging within two weeks of injury
Criterion B	Presence of one of the following speech and/or language deficits: 1. Mutism (inability to speak) or 2. Significant impairment in language as indicated by one or more of the following: reduced phrase length (speech limited to single words or 2- or 3-word phrases), agrammatism, atypical speech rate/rhythm (slowed, gaited, ballistic), and/or dysnomia.
Criterion C	Presence of notable changes in mood/affect characterized by irritability (excessive tearfulness, crying, agitation, or anger) emotional lability (rapid changes in mood) and/or flat affect
Criterion D	Presence of motor dysfunction defined as apraxia (inability to execute purposeful movements on command, despite having the physical capability to perform the movement), ataxia (difficulty coordinating muscle movements), dysmetria (undershoot or overshoot of intended position with the hand, arm, or leg), hypokinesia (abnormally diminished motor activity), and/or hemiparesis (weakness of one side of the body)

## Conclusions

We report a case of an adult patient with ataxia and mutism, presumed secondary to both a previous ischemic stroke of the right cerebellar hemisphere, with evidence of clinical involvement of the contralateral cerebellar hemisphere in the setting of hypertensive urgency, which resolved with treatment over a 24-28 hour period. The cerebellar mutism syndrome was diagnosed clinically with a novel combination of mechanisms creating the characteristic syndrome, which subsequently resolved with correction of the ischemia and hypertension. The acute development of mutism with ataxia in an adult patient localizes to the posterior fossa, and should prompt evaluation for ischemia by imaging and appropriate treatment for potential vascular disease.
